# The granulocyte colony stimulating factor pathway regulates autoantibody production in a murine induced model of systemic lupus erythematosus

**DOI:** 10.1186/ar4208

**Published:** 2013-04-08

**Authors:** Margareta Lantow, Ramya Sivakumar, Leilani Zeumer, Clive Wasserfall, Ying-Yi Zheng, Mark A Atkinson, Laurence Morel

**Affiliations:** 1 Department of Pathology, Immunology, and Laboratory Medicine, University of Florida, Gainesville, FL 32610, USA; 2 Current address: Department of Immunology, University Regensburg, 93042 Regensburg, Germany

## Abstract

**Introduction:**

An NZB-derived genetic locus (*Sle2c2*) that suppresses autoantibody production in a mouse model of induced systemic lupus erythematosus contains a polymorphism in the gene encoding the G-CSF receptor. This study was designed to test the hypothesis that the *Sle2c2 *suppression is associated with an impaired G-CSF receptor function that can be overcome by exogenous G-CSF.

**Methods:**

Leukocytes from B6.*Sle2c2 *and B6 congenic mice, which carry a different allele of the G-CSF receptor, were compared for their responses to G-CSF. Autoantibody production was induced with the chronic graft-versus-host-disease (cGVHD) model by adoptive transfer of B6.bm12 splenocytes. Different treatment regimens varying the amount and frequency of G-CSF (Neulasta^®^) or carrier control were tested on cGVHD outcomes. Autoantibody production, immune cell activation, and reactive oxygen species (ROS) production were compared between the two strains with the various treatments. In addition, the effect of G-CSF treatment was examined on the production autoantibodies in the B6.*Sle1.Sle2.Sle3 *(B6.TC) spontaneous model of lupus.

**Results:**

B6.*Sle2c2 *and B6 leukocytes responded differently to G-CSF. G-CSF binding by B6.*Sle2c2 *leukocytes was reduced as compared to B6, which was associated with a reduced expansion in response to *in vivo *G-CSF treatment. G-CSF *in vivo *treatment also failed to mobilize bone-marrow B6.*Sle2c2 *neutrophils as it did for B6 neutrophils. In contrast, the expression of G-CSF responsive genes indicated a higher G-CSF receptor signaling in B6.*Sle2c2 *cells. G-CSF treatment restored the ability of B6.*Sle2c2 *mice to produce autoantibodies in a dose-dependent manner upon cGVHD induction, which correlated with restored CD4^+ ^T cells activation, as well as dendritic cell and granulocyte expansion. Steady-state ROS production was higher in B6.*Sle2c2 *than in B6 mice. cGVHD induction resulted in a larger increase in ROS production in B6 than in B6.*Sle2c2 *mice, and this difference was eliminated with G-CSF treatment. Finally, a low dose G-CSF treatment accelerated the production of anti-dsDNA IgG in young B6.TC mice.

**Conclusion:**

The different *in vivo *and *in vitro *responses of B6.*Sle2c2 *leukocytes are consistent with the mutation in the G-CSFR having functional consequences. The elimination of *Sle2c2 *suppression of autoantibody production by exogenous G-CSF indicates that *Sle2c2 *corresponds to a loss of function of G-CSF receptor. This result was corroborated by the increased anti-dsDNA IgG production in G-CSF-treated B6.TC mice, which also carry the *Sle2c2 *locus. Overall, these results suggest that the G-CSF pathway regulates the production of autoantibodies in murine models of lupus.

## Introduction

Systemic lupus erythematosus (SLE) is an autoimmune disease with a complex etiology in which the production of pathogenic autoantibodies (autoAbs) results in cellular and tissue damage. Aside from B cells, which produce these autoAbs, and CD4^+ ^T cells, which provide B cell help for the generation of class-switched, affinity maturated autoAbs, essentially every other immune cell subset has been implicated in SLE pathogenesis. The strong genetic basis of SLE is sustained by a large number of polymorphisms that have been identified in recent years through association studies in large cohorts of patients and controls [1]. Mouse models of SLE have been used extensively to study both the cellular and genetic basis of SLE, and overall, the results obtained from these models have largely been validated in SLE patients. In particular, murine models have revealed a large number of SLE susceptibility genes, which are organized in the same three broad pathways: apoptosis and processing of apoptotic debris, toll-like receptor (TLR) signaling and type I IFN pathways, and lymphocyte activation in both SLE patients and SLE-prone mice [2,3]. The genetic analysis of the NZM2410 mouse model has also shown the existence of both SLE-resistance and suppressor genes. Consequently, the SLE-resistant strain C57BL/6 (B6) carries susceptibility genes that were revealed when combined with either other susceptibility genes provided by the NZM2410 lupus-prone genome, or when subjected to a strong immune stimulation [4,5].

The bm12- chronic graft vs host disease (cGVHD) model is a well-defined model of induced lupus in which B6.C-H2^bm12 ^lymphocytes are transferred into H-2^b ^B6 hosts. Within 3 weeks of transfer, mice develop lupus-like phenotypes including lymphocyte activation and anti-nuclear autoAbs, which are dependent on interactions between donor CD4^+ ^T cells and host autoreactive B cells [6]. We have shown that B6.*Sle2c2 *mice, which are B6 mice carrying an NZM2410 (NZB)-derived genomic region on the telomeric potion of the *Sle2 *locus, are profoundly resistant to bm12-cGVHD induction as compared to their B6 congenic controls [5]. Using mixed bone-marrow (BM) chimeras and functional assays, we have shown that *Sle2c2 *suppression is mediated by BM-derived cells, but not by T cells, B cells, or dendritic cells (DCs). We mapped *Sle2c2 *resistance to a short genomic interval that contains *Csf3r*, the gene encoding for the granulocyte-colony stimulation factor (G-CSF) receptor (G-CSFR) [5]. The NZM2410 allele of *Csf3r *(*Csf3r^N ^*as opposed to the B6 allele *Csf3r^B^*) carries a mutation in exon 10 (rs13477964) that results in a S^378^N substitution in its extracellular domain.

G-CSF is an essential factor in the recruitment and function of neutrophils [7]. G-CSFR expression regulates GVHD in mice [8]. Moreover, neutrophils have recently been implicated at multiple levels in the pathology of SLE or related systemic autoimmune diseases [9]. Neutrophils inflict direct damage to the vasculature [10], the kidneys [11] and the skin [12] of lupus patients. They also contribute to SLE pathogenesis through their direct production of type I IFN [13], or through the highly immunogenic neutrophil extracellular traps (NETs) amplifying the production of either type I IFN [14] or IL-17 [15], although these later findings have been refuted in the MRL/lpr lupus-prone mouse model [16]. G-CSF also controls the number of myeloid derived suppressor cells (MDSCs) [17], which are potent suppressors of alloreactive T cell responses in a GVHD model [17]. In MRL/lpr mice, the percentage of MDSCs increases as disease progresses [18], corroborating our findings of an expanded GR1^lo ^MDSC population in B6 mice with established cGVHD [5].

These studies lead us to hypothesize that the N^378^S mutation in the *Csf3r *gene impairs G-CSFR function. This mutation is located in the solvent-exposed part of the receptor in a putative fibronectin III domain outside the solved structure of the protein [5]. It is therefore impossible at this point to predict its functional significance, although it has the potential to affect ligand binding. We hypothesized that the N^378^S mutation is responsible for the differential bm12-cGVHD response between B6 and B6.*Sle2c2 *mice, which we tested by comparing their response to exogenous G-CSF. We reasoned that if bm12-cGVHD resistance in B6.*Sle2c2 *mice was mediated by a gain of function in suppressive cells such as MDSCs, G-CSF treatment would induce bm12-cGVHD resistance in B6 mice. In contrast, if bm12-cGVHD resistance in B6.*Sle2c2 *mice was due to a loss of function or recruitment in inflammatory neutrophils, then G-CSF treatment would alleviate bm12-cGVHD resistance in B6.*Sle2c2 *mice, which would develop autoAbs and lymphocyte activation comparable to that of B6. Here we demonstrate a weaker binding of mouse G-CSF to B6.*Sle2c2 *leukocytes as compared to B6, as well as a defective expansion of B6.*Sle2c2 *myeloid cells and neutrophils in response to *in-vivo *G-CSF treatment. Furthermore, exogenous G-CSF restored bm12-cGVHD responses in B6.*Sle2c2 *mice. Finally, the B6.*Sle1.Sle2.Sle3 *congenic mice that display a full-blown spontaneous lupus as its NZM2410 parental strain carry the *Sle2c2 *locus [19]. It was therefore predicted that G-CSF treatment would accelerate their autoAb production, which we showed in this study with a low-dose treatment. Overall these results suggest that *Sle2c2 *prevents the induction of systemic autoimmunity by impairing the production of inflammatory neutrophils due to a decreased ability of the G-CSFR to bind its ligand.

## Materials and methods

### Mice

The B6.*Sle2c2 *mice carrying an NZM2410 (NZB)-derived interval located between D4Mit11 and D4Mit72 have previously been described [5]. B6 and B6.C-*H2^bm12^*/KhEg (bm12) were originally purchased from the Jackson Laboratory (Bar Harbor, ME, USA). Cohorts of mice within an experiment were sex- and age-matched, but the reported phenotypes were neither affected by sex or age. The NZM2410-derived triple congenic mice B6.*Sle1.Sle2.Sle3 *(B6.TC) have been previously described [19]. All animal protocols were approved by the Institutional Animal Care and Use Committee at the University of Florida.

#### cGVHD induction and G-CSF treatment

bm12-cGVHD was induced according to an established protocol [6]. Briefly, 50 to 80 × 10^6 ^bm12 splenocytes were injected intraperitoneally into 2 to 4 month-old B6 or B6.*Sle2c2 *mice. Serum was collected for autoAb detection by ELISA as previously described [6] at day (d) 0, 7, 14 and 21 after induction. Antinuclear antibody (ANA) stains were conducted on slides containing fixed Hep-2 cells (Inova Diagnotics, San Diego, CA, USA) with mouse sera diluted 1:40, and revealed with fluorescein isothiocyanate (FITC)-conjugated anti-mouse IgG (Southern Biotech, Birmingham, AL, USA) at 1:50 dilution. Staining intensity was calculated with morphometry software (Metamorph, Molecular Devices, Sunnyvale, CA, USA) for a standardized level of green fluorescence averaged on 10 to 20 randomly selected Hep-2 cells per sample. Samples with an average staining intensity > 1 were considered positive. At d21, mice were sacrificed and splenocytes assessed by flow cytometry.

bm12-cGVHD-induced mice were treated with subcutaneous injections of 1.2, 12.0, or 120.0 ug pegylated human G-CSF (Neulasta^®^, huG-CSF, Amgen, Thousand Oaks, CA, USA), all starting at d1 of induction. Some huG-CSF injections were repeated every 7 d resulting in 4 different treatment regimens: one injection of 120 ug (120 × 1), two or three injections of 12 ug (12 × 2 and 12 × 3), and three injections of 1.2 ug (1.2 × 3). Control mice were injected with 5% dextrose as vehicle control. Each experimental group contained at least five recipient mice per treatment per strain, and control groups were performed side by side for each treatment group. Groups of four un-manipulated mice per strain were injected with huG-CSF and blood was collected every 2 to 3 d to compare the dose-response effect of G-CSF of peripheral blood leukocytes (PBLs) between strains. Serum mouse (m) G-CSF was measured by ELISA with reagents from Peprotech (Rocky Hills, NJ, USA) in sera diluted 1:50. B6.TC and B6 female mice were treated with six weekly injections of 1 ug of Neulasta or 5% dextrose starting at 2 months of age and were sacrificed at week 7. Serum was collected every two weeks and anti-dsDNA IgG, anti-chromatin IgG and total IgG were measured as previously described in sera diluted at 1:100 and 1:5000, respectively [6].

### Flow cytometry

Splenocytes or PBLs were blocked with anti-CD16/CD32 (2.4G2), then stained with pre-titrated amounts of the following FITC-, phycoerythrin (PE)-, allophycocyanin-, or biotin-conjugated Abs: CD4 (RM4-5), CD69 (H1.2F3), CD44 (IM7), CD62L (MEL-14) B220 (RA3-6B2), CD86 (GL1), CD80 (16-10A1), CD22.2 (Cy34.1), I-a^b ^(AF6-120.1), CD11b (M1/70), CD11c (HL3), Ly6C/G (GR-1/RB6-8C5) or isotype controls, all from BD Biosciences (San Diego, CA, USA). Biotinylated Abs were revealed by streptavidin-PercP-Cy5a. p-signal transducer and activator of transcription 3 (Stat3) (Y705) staining was performed after fixation and permeabilization (BD Cytofix/Cytoperm kit). G-CSF binding was performed by incubating splenocytes for 1 h with 25 ng of mG-CSF (ProSpec, East Brunswick, NJ, USA) biotinylated with a Biotin XX Microscale protein labeling kit (Molecular Probes, Eugene, OR, USA), detected by flow cytometry with PE-streptavidin. As negative controls, cells were incubated only with PE-streptavidin. ROS production was detected with 1 μM dihydrorhodamine 123 (DHR, Molecular Probes). Cell staining was analyzed using a FACScalibur or LSRFortessa (Becton Dickinson, Franklin Lakes, NJ, USA). At least 30,000 (PBLs) and 50,000 (splenocytes) events were acquired per sample, and dead cells were excluded based on scatter characteristics.

### Gene expression analysis

Splenocytes and BM cells from B6 and B6.*Sle2c2 *mice (three each) were cultured in complete medium at a concentration of 10^6 ^cells/ml with 0.0, 0.1, or 1.0 ug/ml of Neulasta for 8 h. RNA was extracted and cDNA was synthesized as previously described [20]. Expression of *Itgam *and *Mpo *relative to *Gapdh *was quantified by quantitative PCR measuring the incorporation of Sybergreen (Molecular Probes). The primer sequences were: *Itgam *5'-CCC CAA TTA CGT AGC GAA TG-3'and 5'- TGC TGC GAA GAT CCT AGT TG -3'; Mpo 5'- TGC TCT CGA ACA AAG AGG GT-3' and 5'- CTT TGA CAG CCT GCA CGA-3'; *Gapdh *5'- AGC TTG TCA TCA ACG GGA AG -3' and 5'- GTG GTT CAC ACC CAT CAC AA -3'. Results were calculated with the ΔΔ cycle threshold (Ct) method and normalized to the average values for untreated B6 samples.

### Statistics

Statistical analyses were performed with GraphPad Prism5, using the two-tailed unpaired *t-*test or Bonferroni multiple comparison test when multiple groups were compared. Non-parametric statistics were performed when data were not normally distributed. Graphs show mean and standard error of the mean (SEM), and statistical significance is represented as *P < 0.05, **P < 0.01, and ***P < 0.001.

## Results

### B6.*Sle2c2 *leukocytes bind less G-CSF and expand less to exogenous G-CSF than B6

**Figure 1:**
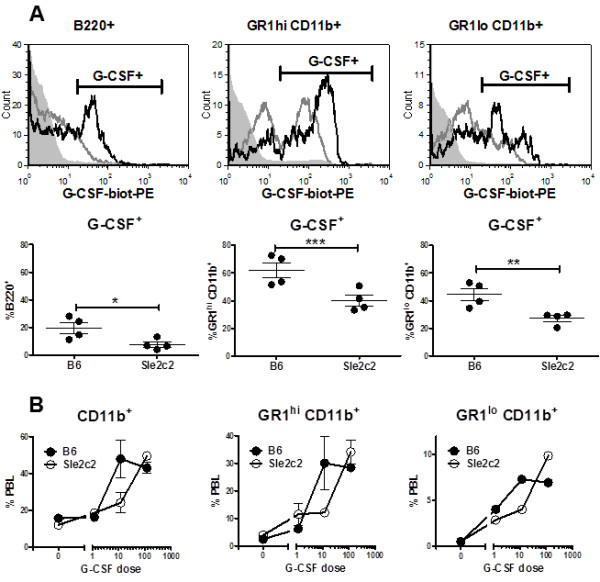
**Differential response of *B6.Sle2c2 *and B6 leukocytes to granulocyte-colony stimulation factor (G-CSF)**. (**A**) Mouse (m)G-CSF binding by B6 and B6.*Sle2c2 *leukocytes measured with biotinylated G-CSF and steptavidin-PE (G-CSF-biot-PE). The top row shows representative overlays of the B6 (black) and *Sle2c2 *(gray) histograms, with the gray-filled histograms showing the controls without added mG-CSF for the indicated gated cell populations. (**B**) Total CD11b^+^, GR1^hi ^CD11b^+ ^and GR1^lo ^CD11b^+ ^peripheral blood leukocytes (PBLs) in untreated B6 and B6.*Sle2c2 *mice (*n *= 6 per strain), and 4 d after injections of 1.2, 12.0 or 120.0 ug human (hu)G-CSF (*n *= 2 per dose per strain). The graphs show mean and standard error of the mean with the significance value from the *t*-test (*P < 0.05; **P < 0.01; ***P < 0.001).

To determine whether the S^378^N mutation in the extracellular domain of the G-CSFR-affected ligand binding, we compared *in vitro *binding of mG-CSF between B6 and B6.*Sle2c2 *splenocytes. As expected, the highest binding was found on GR1^hi ^CD11b^+ ^neutrophils, whereas intermediate levels were found on GR1^lo ^CD11b^+ ^and B cells (Figure 1A). However, every B6.*Sle2c2 *cell subset showed a significantly lower binding of mG-CSF, either as percentage of G-CSF^+ ^cells (Figure 1A), or as the mean fluorescence intensity (data not shown). This was not due to different endogenous levels of G-CSF, since the two strains presented similar serum G-CSF levels (B6, 1.10 ± 0.21 ng/ml; B6.*Sle2c2*, 1.44 ± 0.39 ng/ml, *n *= 20 per strain). To test whether the decreased G-CSF binding by B6.*Sle2c2 *leukocytes provides functional consequences, we compared the effect of *in vivo *huG-CSF treatment on the expansion of myeloid cells between the two strains (Figure 1B). Although human and mouse G-CSF are not identical, a large number of studies, including one by our group [21], have shown that huG-CSF is fully functional in mice. The pegylated form of G-CSF in Neulasta has an extended half-life and a time course analysis showed that the maximum response occurs 4 d after treatment (data not shown). At that time point, a dose-dependent expansion of CD11b^+^, GR1^hi ^CD11b^+ ^and GR1^lo ^CD11b^+ ^PBLs was observed in both strains but a greater amount of hu-G-CSF was required for B6.*Sle2c2 *cells to achieve the level of B6 cell expansion (Figure 1B).

**Figure 2:**
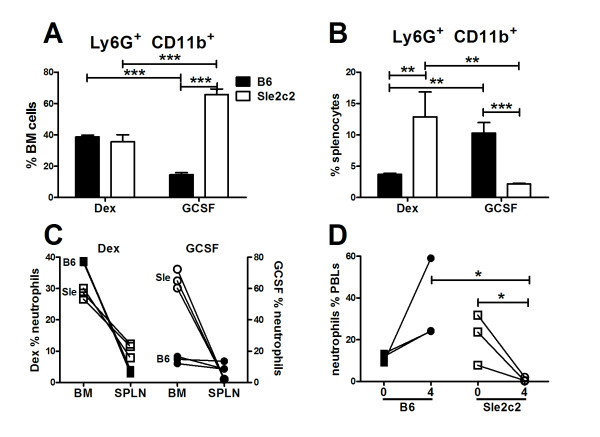
**Differential neutrophil mobilization in response to human granulocyte-colony stimulation factor (huG-CSF) in B6 and B6.*Sle2c2 *mice**. Percentage of Ly6G^+ ^CD11b^+ ^neutrophils present in the bone marrow (BM) (**A**) and spleen (**B**) in B6 and B6.*Sle2c2 *mice 4 d after an injection with 5% dextrose (Dex) or 1 ug hu-G-CSF (GCSF). The graphs show mean and standard error of the mean with the significance of the Bonferroni multiple comparison test performed on three mice per group. The experiment was repeated and the same results were obtained, although with different absolute values (*P < 0.05; **P < 0.01; ***P < 0.001).

One of the functions of G-CSF is to mobilize neutrophils from BM to the periphery [7]. We therefore compared the numbers of neutrophils in the BM and the spleen of B6 and B6.*Sle2c2 *mice 4 d after treatment with G-CSF or dextrose control (Figure 2). As expected, the percentage of neutrophils decreased in the BM of G-CSF-treated B6 mice (Figure 2A and 2C) and increased in their spleens (Figure 2B and 2C). In contrast, the percentage of neutrophils in the BM of G-CSF-treated B6.*Sle2c2 *mice was higher than in the BM of dextrose-treated mice (Figure 2A and 2C), which corresponded to a lack of mobilization of the B6.*Sle2c2 *neutrophils to the spleen (Figure 2B and 2C). The percentages of neutrophils in the blood of these G-GCF-treated mice reflected this difference in mobilization, with an increased percentage of circulating B6 neutrophils but a decreased percentage of circulating B6.*Sle2c2 *neutrophils (Figure 2D).

**Figure 3:**
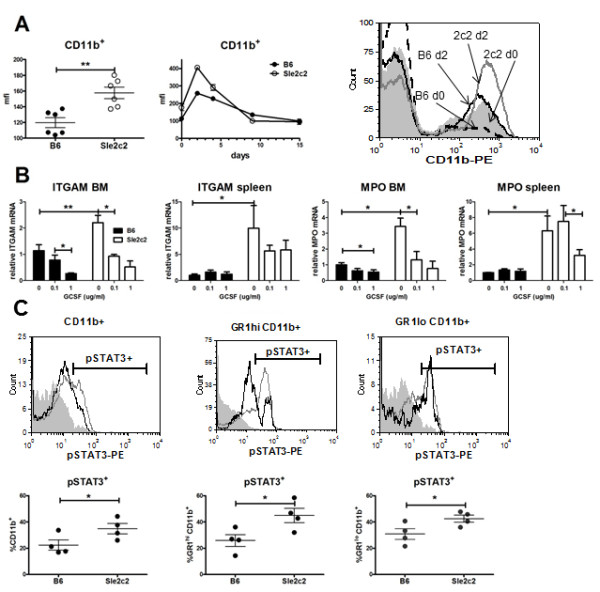
**Differential response of the granulocyte-colony stimulation factor receptor (G-CSFR) target genes in response to G-CSF in *B6.Sle2c2 *and B6 leukocytes**. (**A**) CD11b is upregulated in B6.*Sle2c2 *peripheral blood leukocytes (PBLs) compared to B6 (left panel). Human (hu)G-CSF (120 ug) injected on day (d)1 (arrow) induced a greater CD11b upregulation in B6.*Sle2c2 *than in B6 PBLs (middle panel, *n *= 4 per strain). The overlay histograms on the right show representative CD11b expression in B6 splenocytes at d0 (dash) and d2 (black), and in B6.*Sle2c2 *splenocytes at d0 (filled gray) and d2 (open gray). (**B**) *Itgam *and *Mpo *relative gene expression in bone marrow (BM) and spleen cells cultured for 8 h in medium alone and with 0.1 or 1.0 ug/ml hu-G-CSF. Filled bars represent B6 and open bars represent B6.*Sle2c2 *(*n *= 3 per group). In each graph, the data were normalized to the average of untreated B6 values. (**C**) Intracellular pSTAT3 staining in total CD11b^+^, GR1^hi ^CD11b^+ ^and GR1^lo ^CD11b^+ ^splenocytes (four mice per strain). The top row shows representative overlays of the B6 (open black) and *Sle2c2 *(open gray) histograms, with the filled silver histograms showing the isotype control. The graphs show mean and standard error of the mean with the significance value from the *t*-test (**A **and **C**) or the Bonferroni multiple comparison test (**B**) (*P < 0.05; **P < 0.01; ***P < 0.001).

To determine whether G-CSF signaling differed between B6.*Sle2c2 *and B6 leukocytes, we used three well-known targets, CD11b and myeloperoxidase (MPO) expression and STAT3 phosphorylation [22]. *Ex vivo*, CD11b expression on splenocytes was higher in B6.*Sle2c2 *than in B6 mice (Figure 3A). Treatment with 120 ug of hu-G-CSF significantly increased CD11b expression on CD11b^+ ^PBLs in both strains, but it remained at a higher level on B6.*Sle2c2 *cells. This effect of exogenous G-CSF was abolished by d9 after injection. Similar results were obtained with the expression of CD16/32 (another G-CSF-responsive gene) on CD11b^+ ^PBLs (data not shown). We next compared CD11b/*Itgam *and *Mpo *message expression in BM and spleen cells obtained from B6 and B6.*Sle2c2 *mice 4 d after treatment with 1 ug hu-G-CSF, then treated for 8 h with hu-G-CSF *in vitro *(Figure 3B). In both strains, we observed that hu-G-CSF decreased both *Itgam *and *Mpo *expression. The response pattern was however different between the two strains, with B6.*Sle2c2 *cells being more responsive than B6 at equal doses. In addition, we observed, as for CD11b protein expression after the *in vivo *treatment (Figure 3A), an overall higher expression of *Itgam *and *Mpo *in B6.*Sle2c2 *than in B6 cells. *Ex vivo *STAT3 phosphorylation was also significantly higher in splenocytes from untreated B6.*Sle2c2 *mice; either when considering the percentage of CD11b^+^, GR1^hi ^CD11b^+ ^and GR1^lo ^CD11b^+ ^cells expressing pSTAT3 (Figure 3C), or pSTAT3 mean fluorescence intensity (data not shown). Although factors other than G-CSF signaling may contribute to the differential CD11b expression and STAT3 phosphorylation, the two strains only differ by the *Sle2c2 *interval (< 15 Mb) [5]. While these results demonstrate a lower G-CSF binding and expansion to exogenous G-CSF by leukocytes expressing the *Sle2c2 Cfs3r *allele, evidence of greater G-CSFR signaling in leukocytes expressing the *Sle2c2 Cfs3r *allele was also observed. These apparently paradoxical results should be put in perspective that very little is known about G-CSFR signaling and G-CSFR-responsive genes in primary myeloid cells, as all the studies have been performed with myeloid cell lines (reviewed in [22]). Our results however clearly showed that B6 and B6.*Sle2c2 *myeloid cells respond differently to G-GSF both *in vivo *and *in vitro *by multiple processes, including myeloid/granulocyte expansion, mobilization, and G-CSFR-responsive gene expression.

### Exogenous G-CSF restores cGVHD responses in B6.*Sle2c2 *mice

**Figure 4:**
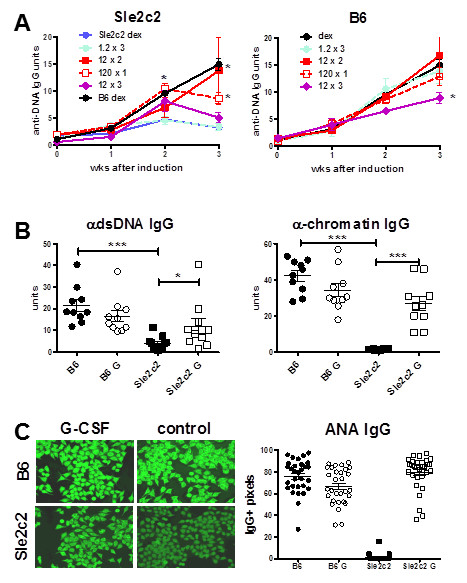
**Exogenous human granulocyte-colony stimulation factor (huG-CSF) restores autoantibody (autoAb) production in B6.*Sle2c2 *mice after induction of chronic graft vs host disease (cGVHD)**. (**A**) huG-CSF-treatment induced anti-dsDNA IgG in B6.*Sle2c2 *mice in a dose-dependent manner (left), but had little effect in B6 mice (right). Five mice per strain per treatment were used. (**B**) Anti-dsDNA and anti-chromatin IgG found at d21 in the combined 120 × 1 and 12 × 2 cohorts. (**C**) Representative ANA IgG staining of Hep-2 cells incubated with sera collected at 21 d in each of the four treatment groups in the 120 × 1 and 12 × 2 cohorts and average staining intensity of 10 to 20 randomly selected cells per sample. Means and standard error of the mean with the significance value from the *t*-test for comparison of treated mice and controls (dex) are shown for each strain for a given time point in **A**, and from Dunnett's multiple comparison test between groups as indicated in **B and C **(*P < 0.05; **P < 0.01; ***P < 0.001).

If the S378N mutation in the G-CSFR is responsible for the cGVHD resistance in B6.*Sle2c2 *mice, we postulated that huG-CSF treatment should eliminate the difference in cGVHD response between B6 and B6.*Sle2c2 *mice. If resistance in B6.*Sle2c2 *mice is due to a G-CSFR loss of function, huG-CSF treatment should restore cGVHD response in B6.*Sle2c2 *mice. On the other hand, if resistance is due to a G-CSFR gain of function, cGVHD resistance would be expected in huG-CSF-treated B6 mice. Based on our time course analysis showing a maximal effect at d4 after injection that was eliminated at d7, we tested a combination of treatment protocols in which the dose (1.2, 12.0, or 120.0 ug) and the frequency (one-, two-, or three-weekly injections starting at cGVHD induction) varied. Two treatment protocols (120 × 1 and 12 × 2) raised the production of anti-dsDNA IgG in B6.*Sle2c2 *mice significantly above the dextrose-treated controls (Figure 4A left), and to a level similar to the B6 controls, especially in the 12 × 2 cohort. For the 120 × 1 cohort, a drop in the third week was observed, possibly because the pharmacological effect of the single huG-CSF treatment was diminishing. With these two treatment regimens combined, the amount of anti-dsDNA and anti-chromatin IgG at d21 was significantly higher in the B6.*Sle2c2 *mice treated with huG-SCF than in controls (Figure 4B). Moreover, the autoAbs produced by treated B6.*Sle2c2 *mice reached equivalent levels as in treated B6 mice. cGVHD induced autoAbs in B6 mice with strong Hep-2 staining that combined cytoplasmic and nuclear patterns (Figure 4C). The 12 × 2 treatment induced Hep-2-staining in B6.*Sle2c2 *mice similar in intensity and pattern as in B6 mice (Figure 4C). Similar results were obtained with the 120 × 1 treatment, although with a more variable intensity (data not shown). The 1.2 × 3 treatment had no effect, indicating that the dose was too low. The 12 × 3 treatment had an intermediate effect in B6.*Sle2c2 *mice, and interestingly was the only regimen that induced a significant decrease in the B6 anti-dsDNA IgG response (Figure 4A right). The other treatments showed a trend in lowering autoAbs produced by B6 mice, but the difference was not significant. Overall, these results demonstrate that exogenous G-CSF eliminates the resistance to cGVHD induction in B6.*Sle2c2 *mice in a dose-dependent manner, with no significant effect in B6 mice at the same dose. This suggests that an impaired G-CSFR response mediates the cGVHD resistance in the B6.*Sle2c2 *strain, which can be compensated by an excess G-CSF to drive equilibrium binding or signaling to the receptor.

#### The restoration of cGVHD response by G-CSF increases CD4^+ ^T cell activation and expands the DC and neutrophil populations

**Figure 5:**
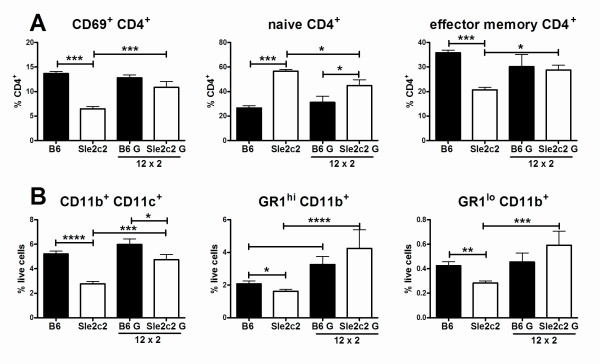
**Restoration of chronic graft vs host disease (Cgvhd)-induced autoantibody (autoAb) by human granulocyte-colony stimulation factor (huG-CSF) correlates with CD4^+ ^T cell activation, as well as DC and GR-1^+ ^cell expansion**. (**A**) huG-CSF 12 × 2 treatment increased CD69 expression, reduced the percentage of naïve CD62L^+ ^CD44^- ^CD4^+ ^T cells and expanded the percentage of the CD62L^- ^CD44^+ ^effector memory CD4^+ ^T cells in B6.*Sle2c2 *mice compared to dextrose-treated controls. (**B**) huG-CSF 12 × 2 treatment increased the percentage of CD11b^+ ^CD11c^+^, GR1^hi ^CD11b^+ ^and GR1^lo ^CD11b^+ ^cells in B6.*Sle2c2 *mice compared to dextrose-treated controls. Means and standard error of the mean are shown with the significance value from Dunnett's multiple comparison tests between groups with five mice per group (*P < 0.05; **P < 0.01; ***P < 0.001).

cGVHD increases the percentage of blasts and the expression of activation markers such as class II MHC, CD22 and CD69 on B6 but not on B6.*Sle2c2 *B cells [5]. Treatment with huG-CSF has minimal effects on these B cell parameters (data not shown). It decreased CD22 expression in a dose-dependent fashion on B6 but not in B6.*Sle2c2 *B cells. However, these changes in B cell activation did not correspond to the observed changes in autoAb production. This suggested that huG-CSF did not restore the cGVHD response through B cells. Host CD4^+ ^T cells are also activated by cGVHD in B6 but not in B6.*Sle2c2 *mice [5]. B6.*Sle2c2 *mice have a significantly greater percentage of splenic CD4^+ ^T cells 3 weeks after cGVHD induction (21.58 ± 0.66% vs 16.35 ± 0.48%, *P *< 0.001). All huG-CSF treatments reduced the size of the CD4^+ ^T cell compartment in both strains, and the 12 × 2 treatment, which was the most successful in restoring a cGVHD response in B6.*Sle2c2 *mice, resulted in an equivalent percentage of CD4^+ ^T cells between strains (17.19 ± 0.55% vs 16.18 ± 0.11%, *P *> 0.05). The 12 × 2 treatment also significantly increased the percentage of CD4^+ ^T cell blasts in B6.*Sle2c2 *mice (controls: B6.*Sle2c2*: 8.54 ± 0.54% vs B6: 16.57 ± 0.75%, *P *> 0.0001; 12 × 2: 14.50 ± 1.26% vs 15.64 ± 1.89%, *P *> 0.05), increased CD69 expression, and reduced the naïve and expanded the effector memory CD4^+ ^T cells in B6.*Sle2c2 *mice (Figure 5A). The similar values in 12 × 2 treated B6 and B6.*Sle2c2 *mice correlated with restored autoAb production in the B6.*Sle2c2 *mice (Figure 4). The percentage of naïve CD4^+ ^T cells was still higher in treated B6.*Sle2c2 *than in treated B6 spleens, yet the difference was smaller than in untreated mice (1.43- vs 2.15-fold). The huG-CSF treatments (12 × 3 and 1.2 × 3) that did not restore autoAbs production in B6.*Sle2c2 *mice had no effect on CD4^+ ^T cell activation (data not shown).

The percentage of CD11b^+ ^CD11c^- ^GR1^- ^macrophages was equivalent between B6 and *Sle2c2 *mice 3 weeks after cGVHD induction. huG-CSF treatments increased the percentage of macrophages in a dose-dependent manner similarly between these two strains without any correlation with autoAb production (data not shown). In contrast, the percentage of CD11b^+ ^CD11c^+ ^myeloid dendritic cells (DCs) was significantly lower in B6.*Sle2c2 *than in B6 mice, and the 12 × 2 treatment significantly expanded this cell population in the spleen of B6.*Sle2c2 *mice to a level similar to that of treated B6 mice (Figure 5B). The percentage of CD11b^+ ^GR1^hi ^neutrophils was significantly higher in un-manipulated B6.*Sle2c2 *than in B6 mice (3.97 ± 0.55% vs 2.50 ± 0.16%, *P *= 0.03). Three weeks after cGVHD induction, this difference was inverted, with B6.*Sle2c2 *values being significantly lower than B6 (Figure 5B). As in the blood of non-induced mice (Figure 1B), 12 × 2 treatment expanded the neutrophil population in the spleen and resulted in equivalent percentages in both strains. Finally, the percentage of CD11b^+ ^GR1^lo ^cells was similar in untreated and unmanipulated B6.*Sle2c2 *and B6 mice (0.49 ± 0.04 vs 0.50 ± 0.02%, *P *> 0.05), but three weeks after cGVHD induction, this population was significantly depleted in B6.*Sle2c2 *mice (Figure 5B). Also as in the blood of non-induced mice (Figure 1B), the 12 × 2 expanded the CD11b^+ ^GR1^lo ^population in B6.*Sle2c2 *mice three weeks after cGVHD induction and resulted in equivalent percentages in both strains (Figure 5B). Overall, these results showed that restoration of the cGVHD response in B6.*Sle2c2 *mice by huG-CSF treatment correlated with an increase in CD4^+ ^T cell activation, and an expansion of the DC, neutrophils and CD11b^+ ^GR1^lo ^cell populations.

### Reactive oxygen species (ROS) production is differentially up-regulated in the cGVHD response between B6.*Sle2c2 *and B6 mice

**Figure 6:**
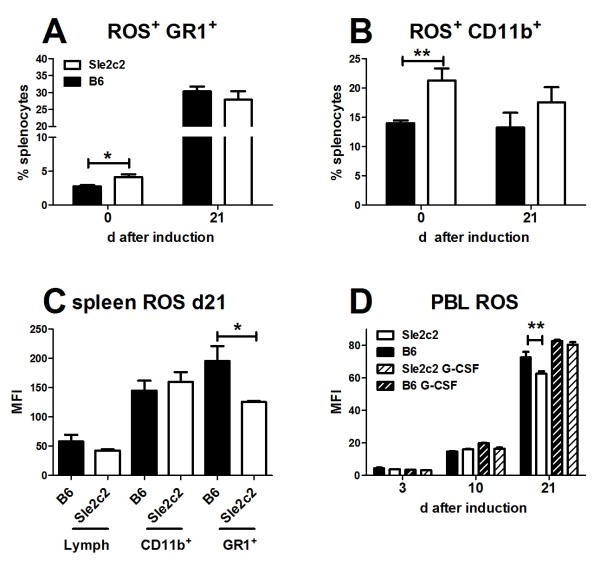
**Reactive oxygen species (ROS) production is differentially upregulated between B6.*Sle2c2 *and B6 mice**. B6.*Sle2c2 *mice present a higher percentage of GR1^+ ^(**A**) and CD11b^+ ^(**B**) splenocytes than B6. Bm12-chronic graft vs host disease (cGVHD) expands the GR1^+ ^ROS^+ ^in both strains, but eliminates the differences between strains. (**C**) At day (d)21 after cGVHD induction the ROS mean fluorescence intensity (MFI) was significantly higher in B6 GR1^+ ^splenocytes than in B6.*Sle2c2*. (**D**) Time course analysis of ROS MFI in total peripheral blood leukocytes (PBLs) during cGVDH induction in mice treated with 12 × 2 human granulocyte-colony stimulation factor (huG-CSF). Means and standard error of the mean for five mice per group, with the significance value from the *t*-test are shown (*P < 0.05; ** *P *< 0.01). These data are representative of two sets of experiments.

**Figure 7:**
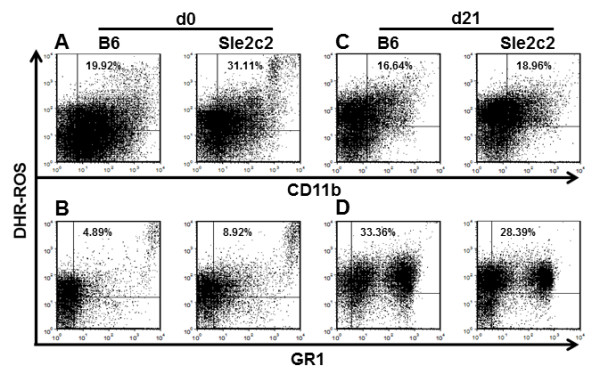
**Representative fluorescence-activated cell sorting (FACS) plots of reactive oxygen species (ROS) production in B6 and B6.*Sle2c2 *splenocytes**. Splenocytes were co-stained for CD11b (A and C) or GR1 (B and D) in un-induced mice (d0) and at d21 after bm12-chronic graft vs host disease (cGVHD) induction. The positions of the quadrants were determined with anti-CD11 and anti-GR1 isotype controls, and with cells stained with surface makers in the absence of the Dihydrorhodamine 123 (DHR) dye. The percentages correspond to double-positive ROS^+ ^CD11b^+ ^or GR1^+ ^live splenocytes.

B6.*Sle2c2 *spleens contain significantly more ROS^+ ^granulocytes cells (Figure 6A and 7B) and myeloid (Figure 6B and 7A) than B6, although no difference was observed for the intensity of ROS expression (mean fluorescence intensity, MFI) on ROS^+ ^cells (data not shown). The same result was obtained with PBL (data not shown). Bm12-cGVHD induction expanded by over 5-fold the population of ROS^+ ^granulocytes in both strains, and eliminated the difference between strains (Figure 6A and 7D). The percentage of ROS^+ ^myeloid splenocytes was also similar between strains after cGVHD induction (Figure 6B and 7C). The ROS MFI was, however, significantly higher on B6 than B6.*Sle2c2 *granulocytes after cGVHD induction (Figure 6C). A time course analysis of ROS expression in B6 and B6.*Sle2c2 *PBLs at d3, 10 and 21 after cGVDH induction demonstrated a robust ROS induction in both strains, but that reached significantly higher levels in B6 than in B6.*Sle2c2 *mice at d21 (Figure 6D). The 12 × 2 treatment induced a higher ROS production in B6.*Sle2c2 *PBLs eliminating the difference with B6 mice, either treated or controls (Figure 6D). Therefore, steady state levels of ROS are higher in B6.*Sle2c2 *than in B6 mice. cGVHD induction greatly upregulates ROS production to a higher level in B6 than in B6.*Sle2c2 *mice, which is corrected by huG-CSF treatment. Taken together, these results suggest that variations in ROS production regulate the cGVHD response and that G-CSF signaling contributes to these variations.

### Exogenous G-CSF accelerates anti-dsDNA IgG in lupus-prone B6.TC mice

**Figure 8:**
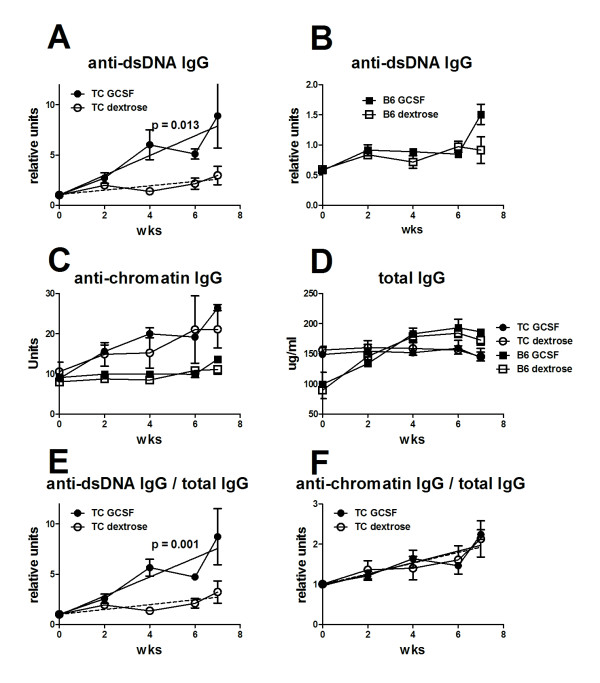
**Human granulocyte-colony stimulation factor (huG-CSF) treatment accelerates anti-dsDNA IgG production in B6.TC mice**. Anti-dsDNA IgG production in B6.TC mice (**A**) and B6 controls (**B**) that were treated weekly with 1 ug huG-CSF (GCSF) or 5% dextrose (dextrose) starting at 2 months of age (*n *= 3 per group). Results for B6.TC mice were normalized to their individual anti-dsDNA IgG levels before treatment (week 0) set as 1. Results for B6 were normalized to the average units of all B6.TC mice before treatment to account for the large difference between B6.TC and B6 anti-dsDNA IgG production. (**C**) Anti-chromatin IgG production in the four cohorts. Unit values are shown because minimal variation was observed before treatment between and within cohorts. The regression lines between the huG-CSF-treated B6.TC and B6 cohorts have significantly different slopes (*P *= 0.005) but not between dextrose-treated cohorts (*P *= 0.17). (**D**) Total serum IgG in the four cohorts. B6.TC mice had significantly more IgG than B6 before treatment (152 vs 95 ug/ml, *P *= 0.001), but that difference disappeared with time. The huG-CSF treatment did not change total IgG production in neither strain. Anti-dsDNA (**E**) and chromatin (**F**) IgG in B6.TC mice normalized to their total IgG production. The autoantibody (autoAb) unit/total IgG ratios were normalized to the individual values before treatment set as 1. The graphs show mean and standard error of the mean at the indicated time points after treatment. For **A **and **E**, the *P*-values correspond to an *F*-test comparing the slopes for the two linear regression lines corresponding to the two treatments.

B6.TC mice first produce anti-chromatin IgG between 2 to 3 months of age then anti-dsDNA IgG between 4 and 5 months of age [19]. The presence of *Sle2c2 *in the B6.TC genome predicts that G-CSF treatment would accelerate their autoAb production as it did in the induced lupus model. We treated 2-month-old B6.TC and B6 female mice with six weekly injections of either 1ug hu-G-CSF or dextrose. It has been shown that neutralizing anti-hu G-CSF antibodies develop with a greater number of injections [23]. G-CSF treated B6.TC mice developed anti-dsDNA IgG significantly faster than control mice (Figure 8A). The G-CSF treatment did not induce anti-dsDNA IgG in B6 mice (Figure 8B). G-CSF, however, had no effect on anti-chromatin IgG, which increased at a similar rate in both treated and control B6.TC mice, and stayed at background levels in treated and control B6 mice (Figure 8C). G-CSF treatment did not affect total IgG levels (Figure 8D) or IgM (data not shown) in either strain, indicating that the effect of G-GSF was specific for anti-dsDNA IgG. This was confirmed when the production of anti-dsDNA IgG was normalized to total IgG (Figure 8E). It was not the case for anti-chromatin IgG (Figure 8F), which may be related to the fact that the anti-chromatin response was already ongoing when the treatment was initiated. Nevertheless, these results show that exogenous G-CSF increased production of anti-dsDNA IgG in mice carrying the *Sle2c2 *locus in a spontaneous model of lupus.

## Discussion

This study was performed to test the hypothesis that the S^378^N mutation in the *Csf3r *gene results in defective G-CSFR signaling and is responsible for the differential autoimmune response between B6 and B6.*Sle2c2 *mice. In our previous study, we speculated that bm12-cGVHD resistance in B6.*Sle2c2 *mice was mediated by enhanced MDSC function or number. However, G-CSF treatment did not induce bm12-cGVHD resistance in B6 mice, but instead eliminated bm12-cGVHD resistance in B6.*Sle2c2 *mice. We therefore conclude that B6.*Sle2c2 *mice are resistant to autoimmunity due to their incapacity to mobilize pro-inflammatory G-CSF-responsive BM-derived cells, most likely neutrophils. Indeed, we report here that B6.*Sle2c2 *leukocytes bind less G-CSF and expand less in response to *in vivo *G-CSF treatment. On the other hand, G-CSFR-responsive genes are expressed at higher levels in B6.*Sle2c2 *leukocytes, which present a greater STAT3 phosphorylation, indicating a higher level of G-CSFR signaling. Although the lower cell expansion to G-CSF and the higher G-CSFR signaling suggest opposing responses, it is possible that the latter is a steady-state compensatory response to the former. This hypothesis will require extensive comparison of G-CSFR signaling between the two alleles, including in cell lines transfected with G-CSFR constructs differing only at S^378^N.

The lower binding of G-CSF and impaired cellular expansion in response to exogenous G-CSF lead us to hypothesize that exogenous G-CSF would compensate for a loss of function phenotype. To this end, we indeed found that two huG-CSF treatment regimens restored the production of autoAbs to a level similar to that found in B6. These treatments also restored CD4^+ ^T cell activation as well as DC and granulocyte expansion in B6.*Sle2c2 *mice to the same levels as in B6. This suggested that these cell populations are effectively modified by higher G-CSF availability and they are involved in the production of autoAbs in this model.

We also found that B6.*Sle2c2 *myeloid cells and granulocytes produced more ROS at steady-state than B6 cells. A recent study found a strong association between a coding mutation in the human *NCF2 *gene with SLE susceptibility, and determined that the disease associated allele was associated with a lower ROS production [24]. This is consistent with decreased levels of *Ncf1*, a gene encoding for another unit of the nicotinamide adenine dinucleotide phosphate-oxidase (NADPH) oxidase complex, being associated with collagen-induced arthritis [25] and experimental allergic encephalomyelitis [26]. Upon bm12-cGVHD induction, ROS production is upregulated to a greater level in the B6 than B6.*Sle2c2 *granulocytes. huG-CSF treatment increased ROS production in B6.*Sle2c2 *mice, which was concomitant with their production of autoAbs. These results suggest that the NZM2410 allele of *Csf3r *results in a higher ROS production at steady state levels of G-CSF that maintains CD4^+ ^T cell tolerance, as it has been shown in other models [27]. We showed here that bm12-cGVHD induction recruits inflammatory neutrophils, as is the case in human SLE [28], of which B6.*Sle2c2 *mice cannot achieve the same expansion as B6 mice unless exogenous G-CSF is provided. Signaling through the G-CSFR is critical for the development and function of inflammatory granulocytes, whereas G-CSFR deficiency is protective in collagen-induced arthritis [29]. We therefore propose that impaired G-CSF binding and G-CSFR signaling is protective again systemic autoimmunity in two phases, first by producing higher levels of ROS, which maintains T cell tolerance, and second by failing to support the expansion of inflammatory neutrophils, which can amplify the autoimmune response either through the type I IFN or Th17 pathways [9].

We have previously reported that there is no difference in *Csf3r *expression between B6 and B6.*Sle2c2 *mice [5]. Genomic sequencing of the *Csf3r *locus has identified a large number of intronic polymorphisms and four coding synonymous mutations, but S^378^N was the only coding non-synonymous mutation identified between the NZM2410 and B6 genomes (unpublished observations). None of the genes located within *Sle2c2 *locus have known functions in the G-CSF pathway. This implies that the differences that we report here between B6 and B6.*Sle2c2 *mice in their response to G-CSF are most likely due to the S^378^N polymorphism. This will, however, need to be formally addressed by stringent structure/function studies.

We have used the bm12-cGVHD model to test the hypothesis that *Sle2c2 *suppression involves the G-CSF pathway, as it is a very robust model that leads to a rapid synchronized production of autoAbs with very little inter-individual variation. However, we have evidence that *Sle2c2 *also suppresses spontaneous autoimmune responses in two NZM2410-derived models [30,31] and we have now shown that a low-dose treatment with G-CSF accelerates the spontaneous production of anti-dsDNA IgG in B6.TC mice, which includes *Sle2c2 *[19].

An association between G-CSF treatment and disease exacerbation is supported by clinical findings, where G-CSF treatment of neutropenic SLE patients can be associated with severe flares [32]. Moreover, depending on the dose, G-CSF treatment can either prevent or accelerate disease in the MRL/lpr mouse model of SLE [33]. While these two reports did not explore the mechanism of disease exacerbation, we propose that it overlaps with the resistance or susceptibility to induced autoAb production mediated by the S378N G-CSFR polymorphism, and that the B6.*Sle2c2 *and B6.TC strains offer an excellent model to identify the cellular and molecular bases on how G-CSF modulates SLE pathogenesis.

Contrary to the results reported here, G-CSF treatment has been reported to promote reversal [21] or prevention [34] of type 1 diabetes in the NOD mouse. In this model, NADPH oxidase impairment through *Ncf1*-deficiency was associated with disease protection [35], contrary to the results obtained in SLE patients with *NCF2-*impaired function [24]. These results suggest an inverse correlation between the protective effect of exogenous G-CSF and the protective effect of the NADPH oxidase products in autoimmune diseases. This has significant implications for devising strategies targeting the G-CSF pathways in opposite direction in diseases such as SLE and type I diabetes.

## Conclusions

Exogenous G-CSF increases the production of autoAb in B6.*Sle2c2 *and B6.TC mice and both of these strains carry the same mutation in the G-CSFR. Furthermore, we have shown a defective mobilization of BM neutrophils in response to G-CSF treatment of B6.*Sle2c2 *mice as well as a different expression of G-CSF-responsive genes between B6 and B6.*Sle2c2 *mice. Overall, these data suggest that mice carrying the G-CSFR mutation are defective in mobilizing and/or activating inflammatory neutrophils, which inhibits the production of autoAbs. Further experiments will be required to demonstrate a direct role of neutrophils in this process.

## Abbreviations

ANA: antinuclear antibodies; autoAb: autoantibody; B6: C57BL/6; BM: bone-marrow; bm12: B6.C-H2^bm12^; cGVHD: chronic graft vs host disease; ct: cycle threshold; DC: dendritic cell; ELISA: enzyme-linked immunosorbent assay; FACS: representative fluorescence-activated cell sorting; FITC: fluorescein isothiocyanate; G-CSF: granulocyte-colony stimulation factor; G-CSFR: granulocyte-colony stimulation factor receptor; huG-CSF: human G-CSF; IFN: interferon; Ig: immunoglobulin; IL: interleukin; MDSC: myeloid-derived suppressor cell; MFI: mean fluorescence intensity; mG-CSF: mouse G-CSF; MPO: myeloperoxidase; NADPH: nicotinamide adenine dinucleotide phosphate-oxidase; NET: neutrophil extracellular trap; PBL: peripheral blood leukocyte; PCR: polymerase chain reaction; PE: phycoerythrin; ROS: reactive oxygen species; SEM: standard error of the mean; SLE: systemic lupus erythematosus; STAT3: signal transducer and activator of transcription 3; TLR: toll-like receptor.

## Competing interests

The authors declare that they have no conflict of interest

## Authors' contributions

ML performed the flow cytometry analysis and the *in vitro *assays. RS performed the neutrophil mobilization assays, the expression assays in G-CSF-responsive genes and the treatment study with lupus-prone mice. LZ performed the cGVHD induction and G-CSF treatments of these mice, antibody measurements and flow cytometry. CW assisted with the conception and implementation of the *in *vivo G-CSF treatment studies. YYZ assisted with the neutrophil mobilization assay. MA participated in the study design and coordination, as well as in data interpretation. LM conceived the study, overviewed data analysis and interpretation, and wrote the paper. All authors read and approved the final manuscript.
